# Eat, drink and gamble: marketing messages about ‘risky’ products in an Australian major sporting series

**DOI:** 10.1186/1471-2458-13-719

**Published:** 2013-08-05

**Authors:** Sophie Lindsay, Samantha Thomas, Sophie Lewis, Kate Westberg, Rob Moodie, Sandra Jones

**Affiliations:** 1 Faculty of Business and Economics, Monash University, Melbourne, VIC, Australia; 2 Centre for Health Initiatives, Faculty of Social Sciences, University of Wollongong, Wollongong, NSW 2522, Australia; 3 Faculty of Health Science, University of Sydney, Sydney, NSW, Australia; 4 School of Economics, Finance and Marketing, RMIT University Melbourne, Victoria, Australia; 5 Melbourne School of Population Health, University of Melbourne, Melbourne, VIC, Australia

## Abstract

**Background:**

To investigate the alcohol, gambling, and unhealthy food marketing strategies during a nationally televised, free to air, sporting series in Australia.

**Methods/approach:**

Using the Australian National Rugby League 2012 State of Origin three-game series, we conducted a mixed methods content analysis of the frequency, duration, placement and content of advertising strategies, comparing these strategies both within and across the three games.

**Results:**

There were a total of 4445 episodes (mean = 1481.67, SD = 336.58), and 233.23 minutes (mean = 77.74, SD = 7.31) of marketing for alcoholic beverages, gambling products and unhealthy foods and non-alcoholic beverages during the 360 minutes of televised coverage of the three State of Origin 2012 games. This included an average per game of 1354 episodes (SD = 368.79) and 66.29 minutes (SD = 7.62) of alcohol marketing; 110.67 episodes (SD = 43.89), and 8.72 minutes (SD = 1.29) of gambling marketing; and 17 episodes (SD = 7.55), and 2.74 minutes (SD = 0.78) of unhealthy food and beverage marketing. Content analysis revealed that there was a considerable embedding of product marketing within the match play, including within match commentary, sporting equipment, and special replays.

**Conclusions:**

Sport is increasingly used as a vehicle for the promotion of range of ‘risky consumption’ products. This study raises important ethical and health policy questions about the extent and impact of saturation and incidental marketing strategies on health and wellbeing, the transparency of embedded marketing strategies, and how these strategies may influence product consumption.

## Background

Elite sport sponsorship has emerged as one of the fastest growing forms of commercial marketing [1]. Overt forms of marketing during sporting events include commercial break advertisements, uniform sponsorship, logos, broadcast sponsorship, sponsored placements, sponsored competitions, boarding and signage, sponsored match replays, and product endorsements [2-7]. Indirect forms of marketing include accessing membership lists, providing uniforms or vehicles to team members, or having sole rights to provide a certain product during a sporting event, such as Heineken’s “sole pouring rights” during the 2012 London Olympics [8,9].

Sports sponsorship creates a range of valuable, and often inseparable, relationships between companies and the sporting teams and codes that they sponsor [6,8,10,11]. Along with broad marketing goals of increased brand awareness [12], sporting events allow industry to align their product with an activity that is perceived as ‘healthy’ and ‘positive’ for the community, thus enabling them to improve perceptions of ‘corporate citizenship’ and perceived community contribution [2,13,14].

Despite calls for the regulation of the marketing of products that may pose short and/or long term risks for the health and wellbeing of some individuals (such as energy dense or unhealthy foods, tobacco, gambling and alcohol products) [2,11] and subsequently defined in this paper as *‘risky products’*, sporting codes regularly argue that their financial viability depends on sponsorship deals (including television broadcast rights) [15]. For example, the mass commercialization of the Olympic Games (after concerns that the games would no longer be financially viable for most host cities) led to a vast increase in sponsorship of products that may have negative health impacts on the community – such as energy dense foods [16]. The International Olympic Committee reportedly received close to USD $1 billion from the top eleven official sponsors (including Coca-Cola and McDonald's) of the 2010 Vancouver Winter Olympics and 2012 London Summer Games [17]. Gambling ‘shirt sponsorship’ deals run into millions of dollars for teams in the English Premier League [18].

In Australia, gambling companies reportedly pay approximately AUD$2 million per season to align themselves with the Australian Football League (AFL) [19]. In 2013, it was reported that Australian bookmaker Tom Waterhouse (TomWaterhouse.com) signed a $50 million, five year deal to become the National Rugby League’s gambling partner, with an additional $15 million deal with Australian free to air television Channel Nine [20]. Carlton United Breweries are reportedly due to sign a $50 million deal to align with the Australian Football League for the next 10 years [21]. Companies pay upwards of USD$3.5million for a 30 second advertising slot during the National Football League ‘Superbowl’ (including products such as Pepsi, Coca-Cola, and M&Ms), which not only attract national but international attention [22].

There has been growing support for the introduction of policies to reduce or restrict the amount of sponsorship for products that may pose health risks within elite sport [2,6,23,24]. In 2013, and partly in response to growing community criticism of the promotion of gambling during Australian sporting matches, the Australian Senate Joint Select Committee on Gambling Reform, established an Inquiry into the promotion of gambling advertising during Australian sport [25]. While the Inquiry is currently ongoing with the Report due in May 2013, a key line of questioning has been about the potential impact of marketing strategies on the wellbeing of children. Similarly, in 2010, the World Health Organisation developed 12 Recommendations aimed at protecting children from the marketing of food and non-alcohol beverages, which were adopted by the World Health Assembly in 2010 [26].

Researchers have also raised concerns about the alignment of *‘risky products’* and sporting events, with research indicating that these associations help to contribute to softening the perception of any risks associated with these products and may negatively impact on health and social outcomes [2,27-32]. Research into the extent of the promotion of tobacco products during sporting matches was important in showing the scope of sports based tobacco promotions [33-35]; and how the tobacco industry used sports based sponsorship to circumvent wider restrictions on the promotion of tobacco products [36,37]. Subsequent regulation of all types of promotions for tobacco products during sporting matches is seen by many as an important step forward in the anti-tobacco movement and in reducing smoking rates, particularly among younger people [35,38].

Most recently, researchers have investigated the sports based marketing of products relating to three complex public health issues: 1) Obesity and nutrition (unhealthy foods and beverages- those products high in saturated fats, *trans*-fatty acids, free sugars, or salt) [2,23] Alcohol misuse and binge drinking (alcohol products and stores) [10,39]; and 3) Problem gambling (most commonly online sports betting products and lotteries) [4,6,10]. A range of studies have explored the role of marketing in encouraging ‘risky’ consumption patterns of unhealthy food, alcohol, and gambling products [32,40,41], including how these marketing strategies are placed within different socio-cultural contexts [32,42,43], how they target different socio-economic and demographic groups [23,44,45], and the impact different types of marketing strategies have on consumption patterns in different groups [46].

A small number of studies have explored the extent and content of alcohol, gambling and unhealthy food marketing during sports matches and the sponsorship of sporting teams. These studies have shown marketing for each of these products is both visible and embedded within sporting ‘match play’. Thomas and colleagues (2012) found there was an average of 50.5 episodes of marketing for gambling products during Australian Football League matches [6]; while Sherriff and colleagues (2010), found that advertising for unhealthy food and alcohol products was visible during 44% and 74% of game footage for three televised professional cricket events [2]. Researchers have also examined the impact of sports based marketing on alcohol and unhealthy food product consumption. For example, researchers have shown that the amount and content of beer advertising during sporting matches influences adolescent vulnerability towards messages about the consumption of beer [47] and that sports sponsorship positively influences children’s perceptions of unhealthy food products and of family purchasing habits [24].

The Australian National Preventative Health Taskforce and the Australian Medical Association have also proposed changes to government policy to reduce people’s exposure to advertising, promotion and sponsorship of energy dense foods and alcohol [48,49]. Some sporting codes have voluntarily chosen to restrict, in particular, alcohol sponsorship and promotion of sport. In 2012, twelve Australian sporting codes joined the ‘Be The Influence’ campaign, which replaced their alcohol sponsorship with government based sponsorship from alcohol taxes to combat alcohol abuse [50]. However, the three major Australian sporting codes – the Australian Football League, National Rugby League, and Cricket Australia did not sign up to the initiative. There have also been smaller shifts in social responsibility practices from individual teams. In 2012 the Australian Football League’s Geelong Cats, and the Australian soccer ‘A’ League’s Melbourne Victory replaced industry based gambling sponsorship with government sponsorship to promote gamblers help services [51,52].

## Methods

### Aims and approach

This study aimed to contribute to existing studies by examining the range of strategies that are used to market three categories of *‘risky products’* – alcoholic beverages; gambling products; and unhealthy foods and non-alcoholic beverages; during three free to air televised sporting events. For the purposes of this paper we defined unhealthy foods and non-alcoholic beverages as those foods and beverages which contained a high amount of added salt/sodium, sugar, or saturated fats.

The study focused on free-to-air coverage of the National Rugby League State of Origin (SOO) 2012 tri-series. The annual SOO involves three matches between two Australian state based National Rugby League teams (the Queensland Maroons and the New South Wales Blues). There is an extensive rivalry between the two teams and, while a domestic competition, the SOO attracts widespread international attention. We selected the SOO 2012 because:

1) Rugby league is rated amongst the top three spectator sports in Australia (along with games in the Australian Football League and Horse Racing) [53];

2) The SOO tri-series have some of the highest television audience ratings of any Australian televised sporting events [54];

3) The three games are played across three Australian states: Victoria, New South Wales, and Queensland, and are shown live on free-to-air metropolitan and regional television; and

4) Both SOO teams have primary sponsorship deals with alcohol companies.

### Data collection and analysis

We digitally recorded the midweek, free-to-air, metropolitan, broadcasts of the three SOO 2012 games (8.30 pm-10.30 pm). Game One, played at Melbourne’s Etihad Stadium (23rd May 2012) attracted a reported national free to air metropolitan viewing audience of 2.51 million [54]; Game Two at Sydney’s ANZ Stadium (13th June 2012), a reported 2.47 million metropolitan viewers and Game Three played at Brisbane’s Suncorp Stadium (4th June 2012), a reported 2.694 million viewers [55]. According to OzTAM figures, young people (aged 5–17) made up 11.5% (290,711 people) of the television audience during Game One; 10.7% (269,499 people) during Game Two; and 11.9% (321,466 people) during Game Three [56].

To document marketing episodes during the games, we adapted a proforma which was previously used to collect data about gambling advertising during Australian Football League games [6]. The proforma was divided into six marketing ‘categories’ and ‘placement areas’: On field; Off Field; Broadcast Sponsorship Lead Ins (textual or narrative information stating that the match broadcast is sponsored by an alcohol, gambling or unhealthy food or beverage product or company); Match Commentary; Commercial Breaks; and on screen ‘Pop Ups’. We defined an episode of marketing as an appearance of a commercial logo, written or spoken narrative which was either visible or audible for at least two seconds. We chose two seconds after a review of tobacco marketing literature, which indicated that repeated, short-term exposure (sometimes referred to as mere exposure) to marketing has a significant impact on an individual’s positive response to a product or brand [57].

Each game was viewed (and reviewed) by a researcher who independently documented the frequency, duration, and content of all alcohol, gambling and unhealthy food and beverage marketing that was clearly visible for at least two seconds. Marketing that appeared simultaneously (for example on jumpers) were counted as separate episodes as long as each episode was visible for at least two seconds. The data collected by these two researchers was discussed to ensure consistency between the researchers – for example in the categorization of products. Where there were discrepancies the footage was viewed again until agreement was reached. We also recorded the exact text or narrative of each of the marketing episodes. In particular we documented any textual or commentary based mention of any alcohol, gambling or unhealthy food or non-alcoholic beverage, the brand names of any specific products, and reference to the consumption of these products or engagement in activities associated with the products.

We used both qualitative and quantitative approaches to analyse the content of the broadcast [58,59]. The duration and episode totals were entered into Microsoft Excel, and descriptive statistics were used to calculate total average number and length of marketing episodes. Qualitative thematic analysis was used to interpret the narratives used within the marketing within each broad product group.

## Results

### Overall episodes and duration

There were a total of 4445 episodes (mean = 1481.67, SD = 336.58), and 233.23 minutes (mean = 77.74, SD = 7.31) of marketing for alcohol, gambling and unhealthy food and beverages during the 360 minutes of televised coverage of the three State of Origin 2012 games. Marketing peaked in Game Three (1736 episodes, 85.92 min); while Game One had the lowest number of marketing episodes (n = 1110), and Game Two had the shortest duration of marketing (71.84 minutes).

### Episodes, duration and content by product type

Table 1 describes product placement for alcohol, gambling and unhealthy food and beverage products during the matches. The following tables provide information about the amount (number of episodes and duration in minutes), type and placement of marketing for alcohol (Table 2), gambling (Table 3), and unhealthy foods and beverages (Table 4).

**Table 1:** Products and placements during State of Origin 2012

**Product**	**Brands**	**On field placements**	**Off field placements**
**Alcohol**	XXXX Gold (Beer)	Fixed banners around stadium	Coach Box
	Victoria Bitter (Beer)	Triangular display boards on field	Towelling
	Tooheys (Beer)	Fixed banner	Backs of chair (dressing room)
	Russian Standard	Try post marker	Drink coolers (exterior and interior)
	Vodka (Spirits)	Goal post padding	Floor signage (dressing room)
	Hahns Superdry (Beer)	Painted logos on field	Wall signage (dressing room)
	Bottlemart (Alcohol store)	Walk through banners	Signage above doors (dressing room)
	Bundaberg (Rum)	Dynamic electronic banners	Locker signage
		Player jersey logos	Scoreboard advertising
		Coach and Staff Uniforms	Player jersey logos
			Coach and team staff uniforms
**Gambling**	TAB Sports Bet	Dynamic electronic banner	None
	Keno Replay	Triangular display board on field	
	Powerball	Keno logo on envelope held by Commentator	
	Tatts Lotto		
**Unhealthy Food and Beverages**	Coca Cola Zero (Beverage)	Triangular display board on field	Scoreboard advertising
	Coca Cola (Beverage)		Dynamic electronic Banner
	McDonalds (Unhealthy Food)	Logos on the match ball	McDonald’s stadium banner
	Dominos (Unhealthy Food)		
	Nutri Grain (Unhealthy Food)		

**Table 2:** Episodes and duration of alcohol marketing during State of Origin 2012 games

**2012 Match**	**Episodes and duration (min)**	**On field**	**Off field**	**Broadcast & commercial lead ins**	**Commentary**	**Commercial break advertising**	**Pop up advertisements**	**Total**
Game 1 Etihad Stadium, Melbourne	Episode (%)*	328 (35%)	581 (61%)	10 (1%)	0 (0%)	5 (1%)	23 (2%)	947
	Duration (%)	13.83 (22%)	44.33 (72%)	0.5 (1%)	0 (0%)	2.5 (4%)	0.57 (1%)	61.73
Game 2 ANZ Stadium, Sydney	Episode (%)	448 (31%)	991 (68%)	7 (0%)	1 (0%)	0 (0%)	2 (0%)	1449
	Duration (%)	17.13 (28%)	44.2 (71%)	0.35 (1%)	0.05 (0%)	0 (0%)	0.33 (1%)	62.06
Game 3 Suncorp Stadium, Brisbane	Episode (%)	734 (44%)	914 (55%)	8 (0%)	1 (0%)	4 (0%)	5 (0%)	1666
	Duration (%)	29.7 (40%)	42.47 (57%)	0.28 (0%)	0.05 (0%)	1.52 (2%)	1.07 (1%)	75.09
Total	Episodes	1510 (37%)	2486 (61%)	25 (1%)	2 (0%)	9 (0%)	30 (1%)	4062
Total	Duration	60.66 (31%)	131 (66%)	1.13 (1%)	0.1 (0%)	4.02 (2%)	1.97 (1%)	198.88

* Percentages are to the nearest whole number.

**Table 3:** Episodes and duration of gambling marketing during State of Origin games

**2012 Game**	**Episodes and duration (min)**	**On field**	**Off field**	**Broadcast & commercial lead ins**	**Commentary**	**Commercial break advertising**	**Pop up advertisements**	**Total**
Game 1 Etihad Stadium, Melbourne	Episode (%)*	105 (77%)	0 (0%)	5 (4%)	12 (9%)	6 (4%)	9 (7%)	137
	Duration (%)	4.07 (40%)	0 (0%)	0.25 (2%)	1.77 (17%)	2.33 (23%)	1.72 (17%)	10.14
Game 2 ANZ Stadium, Sydney	Episode (%)	115 (85%)	0 (0%)	5 (4%)	7 (5%)	3 (2%)	5 (4%)	135
	Duration (%)	4.08 (53%)	0 (0%)	0.25 (3%)	1.2 (16%)	1.25 (16%)	0.85 (11%)	7.63
Game 3 Suncorp Stadium, Brisbane	Episode (%)	27 (45%)	0 (0%)	4 (7%)	14 (23%)	7 (12%)	8 (13%)	60
	Duration (%)	1.43 (17%)	0 (0%)	0.2 (2%)	2.4 (29%)	2.98 (36%)	1.38 (16%)	8.39
Total	Episodes	247 (74%)	0 (0%)	14 (4%)	33 (10%)	16 (5%)	22 (7%)	332
Total	Duration	9.58 (37%)	0 (0%)	0.7 (3%)	5.37 (21%)	6.56 (25%)	3.95 (15%)	26.16

* Percentages are to the nearest whole number.

**Table 4:** Episodes and duration of unhealthy food and beverage marketing during State of Origin games

**2012Match**	**Episodes and duration (min)**	**On field**	**Off field**	**Broadcast & commercial lead ins**	**Commentary**	**Commercial break advertising**	**Pop up advertisements**	**Total**
Game 1 Etihad Stadium, Melbourne	Episode (%)*	5 (31%)	0 (0%)	0 (0%)	0 (0%)	10 (63%)	1 (6%)	16
	Duration (%)	0.1 (3%)	0 (0%)	0 (0%)	0 (0%)	3.3 (91%)	0.22 (6%)	3.62
Game 2 ANZ Stadium, Sydney	Episode (%)	16 (64%)	5 (20%)	0 (0%)	0 (0%)	2 (8%)	2 (8%)	25
	Duration (%)	0.67 (31%)	0.18 (8%)	0 (0%)	0 (0%)	1 (47%)	0.3 (14%)	2.15
Game 3 Suncorp Stadium, Brisbane	Episode (%)	4 (40%)	0 (0%)	0 (0%)	0 (0%)	5 (50%)	1 (10%)	10
	Duration (%)	0.25 (10%)	0 (0%)	0 (0%)	0 (0%)	2.02 (83%)	0.17 (7%)	2.44
Total	Episodes	25 (49%)	5 (10%)	0 (0%)	0 (0%)	17 (33%)	4 (8%)	51
Total	Duration	1.02 (12%)	0.18 (2%)	0 (0%)	0 (0%)	6.32 (77%)	0.69 (8%)	8.21

* Percentages are to the nearest whole number.

#### Alcohol

##### Overview

There were a total of 4062 episodes of alcohol marketing across the three games (mean = 1354, SD = 368.79), with a total duration of 198.88 minutes (mean = 66.29 min, SD = 7.62). Game Three had the greatest amount of alcohol marketing with 1666 episodes, lasting 75.09 minutes. Game One had the least amount of marketing with 947 episodes, lasting 61.73 minutes. Beer was the most frequent alcohol product marketed across all games, with marketing for four beer brands: XXXX Gold, Victoria Bitter, Tooheys and Hahn SuperDry. Five spirit products were marketed, Canadian Club, Jack Daniels Tennessee Honey Whisky, Russian Standard Vodka and Bundaberg Rum; but were marketed less frequently than beer. In Games One and Two, Bottlemart, a chain of Australian liquor stores was also advertised during the commercial breaks.

##### On field marketing

There were a total 1510 episodes (37%) of ‘On Field’ marketing (mean = 503.33, SD = 208.58). Marketing for alcohol was placed in a diverse range of spaces on the field including: fixed banners around the playing field for Victoria Bitter, Tooheys, Bottlemart, XXXX Gold, and Bundaberg Rum; dynamic electronic banners around the field for Victoria Bitter and Bundaberg Rum; triangular display boards on the field for Bundaberg, Victoria Bitter, XXXX Gold, and Tooheys; padding surrounding the goal posts for XXXX Gold; painted logos on the field for Victoria Bitter and XXXX Gold; and run-through banners as the players exited the changing room to the field for Victoria Bitter. The most continuously visible marketing occurred on player uniforms. The XXXX Gold logo was placed in six different locations on the Queensland Maroons player uniform (on either side of the jersey sleeves, the front left jersey corner, across the upper back of the jersey, and on either side of the shorts). The Victoria Bitter logo appeared in two places on both the front and back of the New South Wales Blues player uniform, with the slogan “*The best cold beer*” also written on the back of the jersey.

##### Off field marketing

There were 2486 episodes (61%) of ‘Off Field’ marketing (mean = 828.67, SD = 217.91). These included: at the base of the Queensland coaching box during each game for XXXX Gold and Victoria Bitter; and in team dressing rooms on XXXX Gold logo’s on towels, the back of chairs and drink coolers, and signage above dressing room doors, Victoria Bitter logos on each player locker, and dual Victoria Bitter and XXXX Gold logos on floors and walls. During pre-match and half time break coverage, marketing was visible during dressing room interviews with coaches; and footage of team warm-ups. One of the most embedded and overt forms of off field marketing occurred at half-time, when players sat in a circle around a prominent painted logo for either XXXX Gold (Queensland Maroons) or Victoria Bitter (NSW Blues), while they received a talk about play from the coach. During match play, dressing room marketing was also visible during ‘sin bin’ coverage (when a player was directed to spend ten minutes of game play in the dressing room for rough conduct).

##### Commentary

A number of times during the games, the match commentators specifically linked the game, players and teams to alcohol sponsorship: *“Brett Stewart on his own 30 metre line…on the XXXX sign, sponsors of the Maroons”.* During Game Three commentators made repeated reference to the “*XXXX Gold centre line*”, which featured a logo with the text “*XXXX Island*”, and the *“XXXX Island”* competition (http://www.xxxxisland.com.au/). Commentators outlined the prize and entry requirements, adding that individuals would be able to drink the sponsor’s product on the Island: *“…there might just be a bit of XXXX on that Island, just to make things nice.”*

##### Commercial breaks

Commercial break advertising for alcohol incorporated upbeat music, glamour and humour. Advertisements targeted a young male audience. For example a Tooheys New ‘Supporting Mateship’ commercial used humour to convey a key message that drinking Tooheys New was a way to establish a close bond with a step-brother. The commercial showed two men renovating a house together, and focused on trust and mateship, with the two men ultimately bonding by drinking Tooheys beer in pub [60]. A Canadian Club whisky commercial [61] used glamour and humour to target youth to switch from beer to Canadian Club whisky. The commercial portrayed beer drinkers as old, overweight, and depressed, and contrasted them with a young attractive male at a bar drinking a Canadian Club with a tagline: “…*every time you buy a Canadian Club, a beer fairy dies*”.

##### Screen based pop ups

‘Pop up’ advertisements for Tooheys New appeared during Game One with the text: “*A mid-game fridge run turns a mate of a mate into a mate*”. ‘Pop up’ advertisements for the *‘XXXX Island’* competition also flashed onscreen during game play stating: *“Win a trip to XXXX Island, see wwos.com.au/xxxxisland for details. 18+ only”.*

#### Gambling

##### Overview

There were a total of 332 episodes of gambling marketing across the three games (mean = 110.67, SD = 43.89). The highest number of episodes was in Game One (n = 137), and the lowest in Game Three (n = 60). The total duration of gambling marketing was 26.16 minutes (mean = 8.72, SD = 1.29). Four gambling products were marketed: TAB Sportsbet – an online and retail betting provider offered by Tabcorp; Tatts Powerball – a form of lottery offered by the Tatts Group (a Victorian gaming company); Keno offered by Tabcorp– a numbers game available at 3,457 licensed venues in Victoria, Queensland and New South Wales; and Tatts Lotto - offered by the Tatts Group.

##### On field marketing

There were 247 episodes of ‘On-Field’ marketing across the three games (mean = 82.33, SD = 48.18). This included dynamic electronic banners around the field for Keno and TAB Sportsbet, and triangular shaped display boards positioned around the periphery of the field for TAB Sportsbet.

##### Off field marketing

There was no off field marketing documented for gambling products.

##### Commentary

There were 33 episodes of ‘commentary’ marketing (mean = 11, SD = 3.61). In-game commentary about gambling included commentators announcing the ‘live odds’ associated with the game, and the telephone number to call to back their *“favourite player”* by placing a bet during the game (in Australia live or ‘in the run’ betting is prohibited online, and this type of betting could only be made by making a call to the betting agency). Before the half time break of each game, the match commentators embarked on a discussion of the game statistics, which including an overview of the updated betting odds. During this thirty second segment, the match commentators introduced and crossed to a representative from the online gambling company TAB Sportsbet. Speaking from the field, and using a microphone, appearing similar to a member of the commentary team, the representative used rapid fire betting language to describe the betting odds, and exotic bet options such as who would score the next try. The narrative within this commentary highlighted those engaging in multiple betting options, and that they could bet live during the game:

“Well courtesy of that try right on half time, Queensland now favourites in Origin Two at $1.75. New South Wales $2.10 outsiders. We’ve got a 1.5 point line. I can tell you, the over under are at 27.5. Betting live on a stack of markets including the man of the match? Here’s one for you at half time. Are we going to get home on a wing and a prayer? New South Wales Jarryd Hayne or Akuila Uate to score a try in the second half at $4. No matter where you’re watching the telecast on Nine, bet live at TAB Sportsbet on 133 390”

At times the commentary team also introduced gambling discourses into their call of the game. For example, after the first try was scored in Game One, one match commentator stated: “*Find the ball, find the line, find your bookie! *”

##### Commercial breaks

Paid commercial break advertisements (n = 16) included ‘market updates’ for TAB Sportsbet. These commercials gave the most up-to-date betting odds for the game before and during each game. For example, in one commercial screened during Game One a young, blonde, female representative from TAB Sportbet appeared in a room set up to appear like a financial traders’ room. Computers and staff members appeared in the background as the young woman described the odds in a TAB ‘Market Update’. Statements about problem or responsible gambling are required by law within gambling commercials in Australia (although the exact message and the presentation of the message differs in different jurisdictions). Toward the end of the commercial a small message appeared at the bottom of the screen asking gamblers to think about their choices and with the numbers and website addresses for state based gambling help services.

##### Screen based pop ups

‘Pop up’ advertising (n = 22) for Keno and Tatts Lotto appeared next to the televised score repeatedly throughout each game and were regularly reinforced by commentary. For example “*Keno Replay*” logo flashed on the screen when there was a game replay, and commentators verbally linked these replays to the Keno product: “*…the Keno replay showed Hodges scoring in the corner there*”. A ‘Man of the Match’ competition “Clean up with Keno” also popped up onto the screen, and encouraged the audience to pick the best player of each game, while the commentary team stated: “*…don’t go away, we will be back with the Keno Clean Up, Man of the Match*”. This post-match monetary prize was awarded to the SOO player deemed the “Man of the Match” on the field by a Commentator who held an envelope with a Keno logo.

#### Unhealthy foods and non-alcoholic beverages

##### Overview

There were a total of 51 episodes of marketing for unhealthy food and non-alcoholic beverages across the three games (mean = 17, SD = 7.55). Most episodes were during Game Two (n = 25), with the least during Game Three (n = 10). The total duration of marketing was 8.21 minutes (mean = 2.74, SD = 0.78).

Twelve unhealthy food and beverage brands were marketed: Pizza Hut, KFC, Domino’s Pizza, Coca Cola, McDonald’s, Kelloggs Nutri-grain, Kit Kat, Cadbury, Krispy Kreme Doughnuts, Hungry Jacks, Smith’s Chips and Subway.

##### On field marketing

‘On field’ marketing (n = 25, mean = 8.33 SD = 6.66), accounted for 49% of all unhealthy food and beverage marketing episodes. Unhealthy food and beverage marketing appeared in a range of on field spaces. This included logos for Coca Cola and Nutri-Grain on the match ball; and a triangular display boards on the field.

##### Off field marketing

Off field advertising for unhealthy foods and beverages only occurred during Game Two, and included scoreboard advertising for Coca Cola; dynamic electronic banners featuring advertisements for Coca Cola and McDonalds; and fixed sideline banners around the edge of the stadium for McDonalds.

##### Commentary

There was no commentary based marketing recorded for unhealthy foods and beverages.

##### Commercial breaks

About a third of all marketing content for unhealthy foods and beverages was via commercial break advertising (n = 17, mean = 5.67, SD = 4.04). However, commercial break advertising accounted for the vast majority (77%) of the total duration of unhealthy food and beverage marketing. Often these commercials demonstrated an association between unhealthy food and sport. For example, Domino’s pizza delivery service advertising featured a group of young people visiting a friend’s lounge room setting to watch “*the big event*” and included the sound effects of a sports umpire whistle [62]. McDonald’s advertising was also sporting based, and promoted their Olympic Games “*Atlanta Pork McRib*” product by featuring two men dressed in sporting attire watching televised coverage of an American athlete setting a world record, and the audio transcript:*“…the new Atlanta pork McRib. Race into Macca’s now, before they run out”.* The commercial ended with the McDonald’s logo shown adjacent to the Olympic rings with the text “*proud partner*” below the two logos [63].

##### Screen based pop ups

Pop up advertising for KFC appeared on screen as well as a KFC sponsored *“Impact Player”* marketing pre-game segment during Games One, Two and Three. This segment occurred for 13 seconds per game and featured a KFC *“Impact Player”* logo before introducing the *“Impact Player”* of that specific game followed by a statement referring to the player such as: *“Cooper Cronk, coming off the bench, He’s tonight’s KFC Impact player”*. Replay footage of the ‘*Impact Player’* was accompanied by a rotating KFC ‘pop up’ logo, which appeared in the top left-hand corner of the television screen.

## Discussion

This study develops on the findings of previous studies because it: a) explores the range of ‘risky’ products marketing within sporting matches; b) looks at how the marketing of these products may vary; and c) provides the first detailed examination of the promotion of these products during the National Rugby League SOO series. While it is important to note that this study did not aim to assess exposure, nor did it aim to examine how marketing actually impacted on the consumption patterns of each of the products under investigation, these are important areas for future investigation, and will add additional evidence to guide policies and interventions. If, like tobacco [64], sports based marketing is particularly instrumental in young people’s experimentation with alcohol, gambling and unhealthy foods and beverages, there may be more compelling reasons to consider the regulation of these products during matches with large child audiences. Studies which directly examine the impact of above and below the line sports based marketing promotions on vulnerable populations are urgently needed.

In this study we observed that marketing for the three products saturated the game coverage. For about two thirds of the television broadcast, the marketing for at least one ‘risky’ product, and most notably alcohol, was visible. While traditionally marketing on free-to-air television is associated with paid commercial break advertisements, this type of marketing accounted for less than 1% of episodes, and just over 7% of the duration of marketing. Understanding the impact of these alternative forms of marketing that are integrated into the match poses a number of challenges for policy makers and public health professionals. Research shows that most consumers understand the persuasive role of advertising and regard it with a degree of skepticism and even disdain [65]. However, with the rise in digital recording devices and paid ‘commercial free’ television, marketers are increasingly searching for innovative ways in which to promote and integrate their brands into programming, in this case sporting match broadcasts [66]. However, similar to product placement in television shows, movies and video games, brands or promotional messages which are integrated into the sporting match may not be subject to the same level of scrutiny or cognitive processing from audiences as occurs with traditional advertising [67]. As such, viewers may not activate the same defense mechanisms against persuasion that they are likely to enlist with advertisements during commercial breaks [66]. While commercial break advertising delivers a clearly defined message in a controlled environment where it is pre-determined where and when the advertisement will appear, this type of marketing is not interactive and although the viewer is exposed to the brand, it does not *“become part of the fans’ experience”*[68]. In contrast, sponsorship provides multiple opportunities and spaces for exposure with some arguing that the emotional power of the sporting event creates a ‘heart/mind’ connection with audience members [2].

Within the SOO games, there was a clear integration between the promotion of in particular alcohol and gambling products and the match play. The distinct alignment of the two beer brands as the main sponsors of each of the teams created a symbolic additional layer of competition within the sporting match. A variety of visual and verbal mechanisms were used to link products and brands with the game. First the frequent short visual ‘bursts’ of marketing for alcohol products; research has shown that this type of sponsorship is more effective than commercial break advertising in influencing positive brand attitudes, and particularly brand familiarity through the use of repeated exposures to the product [69,70]. Visual cues were also regularly supplemented with verbal commentary based cues. Verbal cues may signal a tacit endorsement of the products or brand as well as a blurring of boundaries between the advertiser and sport by incorporating the brand with key elements of the game, thus providing the products with more credibility within the sporting context.

This study also raises questions about the transparency of marketing strategies within sporting matches. Verbal cues may be particularly confusing for audience members – particularly given that gambling metaphors were used by the commentary team – with little clarity about whether the announcements are spontaneous and reflect the view of the commentator or are paid commercial endorsements. Many of these verbal cues also played upon audience members’ emotional attachment to players, teams, and the game itself. For example, in-match commentary, particularly for gambling products played upon this connection, encouraging individuals to back their favourite player or team through live bets during the game. Policy makers have tried to reduce the impact of a range of types of sports sponsorship by introducing legislation to restrict paid commercial advertising [71]. While the Australian Government has signalled that it will work with commercial and subscription broadcasters to reduce and control the promotion of live gambling odds, including banning sporting commentators from mentioning lives odds and banning all live odds during promotion during playing – to date, no legislation has been passed to restrict and reduce this type of marketing. Furthermore, this study provides evidence that industry may be employing a far broader and possibly more influential range of strategies to promote their products during matches. The influence of these strategies on the consumption patterns of different groups will be an important area for further investigation.

The media plays a key role in consumer socialization – that is the process by which young people acquire skills, knowledge, values and motives pertaining to their ultimate role as consumers, even though actual consumption decisions may take place in the future [72]. Whilst the consumption of gambling and alcohol products are restricted to those over 18 years of age, approximately 10-12% of the 2012 State of Origin audience were young people. This raises important ethical and health policy questions about the promotion of potentially ‘risky’ products during sporting matches, and how these strategies may influence young people’s attitudes towards and consumption of these products. It has been suggested that by around the age of five, the majority of children are able to differentiate between programming content and television commercials, although they may not be able to understand the persuasive intent of advertisements until they are seven or eight years of age [73]. However, the commercial intention behind sponsorship is conceptually less well understood by children of a younger age. Only when they reach the age of about twelve do they understand the role of sponsorship in influencing consumption attitudes and behaviour [74]. While alcohol and gambling should be inaccessible to young people, published and anecdotal evidence suggests that young people are still consuming these products [45,46,75]. While previous studies have shown that children have a high awareness of alcohol products and brands during sporting matches, and in particular have a preference for products endorsed by celebrities [41,76][77], what is less clear is how different types of strategies may stimulate the current and long term consumption patterns of these products by young people. Some studies suggest that boys are more likely than girls to exhibit ‘fan’ behaviour and respond to sponsors’ products, while others suggest that the consumption patterns of girls are more influenced by sports celebrity endorsement [78-80].

## Conclusion

Further research and policy analysis should explore the influence that a variety of sports based marketing strategies may play in normalizing and encouraging the consumption of these products for young people. Importantly, researchers and policy makers should also consider the extent to which sporting organisations and related industries may resist restrictions on sponsorship claiming that they impact too substantially on their financial viability. This must include considering a range of different strategies to encourage and incentivise sporting codes to shift their sponsorship alliances.

## Competing interests

The authors have no competing interests to declare.

## Authors’ contributions

SL: PhD student. Collected and analysed data, and contributed to the drafting and critical revision of the manuscript. ST: Conceived this study and the design of the study. She participated in the analysis and interpretation of data, the drafting of the manuscript, and the critical and theoretical revision of the manuscript. SL: Participated in the collection and analysis of data, the original drafting of the manuscript, and the critical revision of the manuscript. KW: Contributed to data interpretation, and the drafting and critical revision of the manuscript. RM: Contributed to data interpretation, and the drafting and critical revision of the manuscript. SJ: Contributed to the data interpretation, and the drafting and critical revision of the manuscript. All authors read and approved the final manuscript.

## Pre-publication history

The pre-publication history for this paper can be accessed here:

http://www.biomedcentral.com/1471-2458/13/719/prepub
